# Incidence and Risk Factors for Postpartum Hemorrhage: A Case-Control Study in a Tertiary Hospital in Greece

**DOI:** 10.3390/medicina59061151

**Published:** 2023-06-15

**Authors:** Kyriaki Mitta, Ioannis Tsakiridis, Themistoklis Dagklis, Riola Grigoriadou, Apostolos Mamopoulos, Apostolos Athanasiadis, Ioannis Kalogiannidis

**Affiliations:** Third Department of Obstetrics and Gynecology, School of Medicine, Faculty of Health Sciences, Aristotle University of Thessaloniki, 541 24 Thessaloniki, Greece

**Keywords:** postpartum hemorrhage, causes, epidemiology, incidence, management, risk factors

## Abstract

*Background and Objectives:* Postpartum hemorrhage (PPH) is an obstetrical emergency and although the mortality rate from PPH has decreased, it is still considered a challenge in obstetrics. This study aimed to estimate the rate of primary PPH, as well as to investigate the potential risk factors and management options. *Material and methods:* This was a retrospective case-control study of all cases with PPH (blood loss > 500 mL, irrespective of the mode of delivery) managed in the Third Department of Obstetrics and Gynecology, Aristotle University of Thessaloniki, Greece, from 2015 to 2021. The ratio of cases to controls was estimated to be 1:1. The chi-squared test was used to examine if there was any relationship between several variables and PPH, while subgroup multivariate logistic regression analyses of certain causes of PPH were also conducted. *Results:* During the study period, from a total of 8545 births, 219 (2.5%) pregnancies were complicated with PPH. A maternal age > 35 years (OR: 2.172; 95% CI: 1.206–3.912; *p* = 0.010), preterm delivery (<37 weeks) (OR: 5.090; 95% CI: 2.869–9.030; *p* < 0.001) and parity (OR: 1.701; 95% CI: 1.164–2.487; *p* = 0.006) were identified as risk factors for PPH. Uterine atony was the main cause of PPH in 54.8% of the women, followed by placental retention in 30.5% of the sample. Regarding management, 57.9% (n = 127) of the women received uterotonic medication, while in 7.3% (n = 16), a cesarean hysterectomy was performed to control PPH. Preterm delivery (OR: 2.162; 95% CI: 1.138–4.106; *p* = 0.019) and delivery via a cesarean section (OR: 4.279; 95% CI: 1.921–9.531; *p* < 0.001) were associated with a higher need for multiple treatment modalities. Prematurity (OR: 8.695; 95% CI: 2.324–32.527; *p* = 0.001) was identified as an independent predictor for an obstetric hysterectomy. From the retrospective analysis of the births complicated by PPH, no maternal death was identified. *Conclusions:* Most of the cases complicated with PPH were managed with uterotonic medication. An advanced maternal age, prematurity and multiparity had a significant impact on the occurrence of PPH. More research is needed on the risk factors of PPH, while the establishment of validated predictive models would be of value.

## 1. Introduction

Postpartum hemorrhage (PPH) is an obstetric emergency that complicates 1–10% of all deliveries and requires appropriate training for effective prevention, recognition and management [1]. To date, there is inconsistency in its definition among evidence-based guidelines around the world. Thus, the Society of Obstetricians and Gynaecologists of Canada (SOGC) defines PPH as blood loss >500 mL following vaginal delivery or >1000 mL after cesarean delivery, whereas the World Health Organization (WHO) defines PPH as blood loss ≥500 mL regardless of the mode of delivery; the American College of Obstetricians and Gynecologists (ACOG) sets the threshold at 1000 mL [2].

The most common causes of PPH are classified using the acronym of the four Ts (tone, trauma, tissue and thrombin); uterine atony accounts for about 70% of PPH cases, genital tract trauma accounts for 15–20% of cases, retention of the placenta and/or membranes increases the incidence of PPH by 3.5 times (10–40%) and coagulation disorders, both inherited and acquired, account for approximately 1% of PPH [3,4,5]. Known risk factors for PPH are prolongation of labor, multiple gestations, multiparity, fetal macrosomia, operative vaginal delivery, uterine ruptures, placental abruption, uterine inversions and abnormal placentation [6,7,8].

Despite the progress in obstetrics, PPH continues to represent the leading cause of maternal morbidity and mortality in many countries around the world [9]. Furthermore, there is a geographic variation in the incidence of PPH; it has been reported to be higher in Africa, North America and Asia [10]. According to a study from China, the incidence of PPH was 0.81% and the two major risk factors were placenta previa and accreta [11].

Taking into consideration the discrepancies in the availability of resources and the differences among populations and cultures, epidemiological data from different countries may be useful. This study aimed to perform a descriptive analysis of cases complicated by PPH, including its management and potential risk factors.

## 2. Materials and Methods

This was a retrospective case-control study conducted at the Third Department of Obstetrics and Gynecology, School of Medicine, Faculty of Health Sciences, Aristotle University of Thessaloniki, Greece, during a 7-year period (January 2015 to December 2021). This department is a tertiary referral center for high-risk pregnancies in Northern Greece.

Based on the definition recommended by the WHO, PPH was defined as blood loss of more than 500 mL, independent of the mode of delivery (vaginal or cesarean) [2]. Blood loss was estimated by weighing the packs and sponges used; 1 milliliter of blood weighs approximately 1 g. The gravimetric method (weighing of the pre- and post-procedure gauze) was the main method of blood loss estimation during the study period. Despite its time-consuming nature, it was always used, especially in severe PPH (>1000 mL blood loss, estimated by a visual estimation).

All singleton pregnancies complicated by PPH were recorded on a datasheet file. The medical history, comorbidities and medication were recorded as well. Those with missing data from their medical history were excluded from the study. Furthermore, regarding those who were receiving medication that may have increased the risk of bleeding (i.e., low molecular-weight heparin or aspirin), we took into consideration the medication as a risk factor and cause of PPH only when there was a laboratory diagnosis of bleeding disorders or when there was no other obvious cause of PPH. The inclusion criteria of the study groups were singleton pregnancies with PPH, irrespective of age, parity, method of conception and medical treatment. The exclusion criteria were missing data from the medical history and multiple (twins or triplets) pregnancies. As for the control group, to minimize selection bias, it was randomly chosen from the same department, meaning that the population was the same as the control group. Moreover, in order to avoid any significant changes in the population characteristics and the management of PPH over time, the control group was selected equally each year during the 7-year period. Specifically, the control group was selected from the first shift performed every three months during the study period. The ratio of cases to controls was 1:1.

The overall incidence and the causes of PPH as well as its management were described, then a case-control analysis was performed on the association of PPH with several variables, including maternal age (>35 vs. ≤35 years old), prematurity (≥37 vs. <37 weeks), parity (nulliparous vs. parous), mode of delivery (vaginal vs. cesarean delivery) and onset of labor (no labor vs. spontaneous onset vs. induction of labor). Furthermore, subgroup analyses of different management options of PPH were conducted. Specifically, we compared the cases of PPH that were managed conservatively (pharmaceutically) with those needing additional management options. Furthermore, we analyzed cases of PPH that required an obstetric hysterectomy with those not requiring a hysterectomy. A comparison of cases of PPH that needed a blood transfusion with those that did not was conducted as well. The aim of these subgroup analyses was to identify any potential risk factors of PPH not responsive to conservative (pharmaceutical) management, obstetric hysterectomies and blood transfusions. The study was conducted according to the STROBE statement [12].

### 2.1. Statistical Analysis

Qualitative variables were described as frequencies (n) and proportions (%). The chi-squared test was employed to assess the associations of maternal age, preterm delivery, parity, onset of labor and mode of delivery with PPH. Additionally, using significant variables (*p* < 0.05) from the univariate analysis, multivariate logistic regression models (enter method) were used to identify independent predictors for conservative management as well as obstetric hysterectomies and blood transfusions in cases of PPH. The odds ratios (ORs) with 95% confidence intervals (CIs) were estimated. A statistical significance was considered to be a *p*-value < 0.05. The statistical program SPSS (IBM SPSS Version 28.0) was employed for the analyses.

### 2.2. Ethics

The women consented to the anonymity of their data and the possible use for research purposes; no incentives were provided. The study was approved by the ethics committee of the Aristotle University of Thessaloniki (63151/29-12-2022).

## 3. Results

From a total of 8545 deliveries during the study period, 219 (2.5%) cases with PPH were identified. Factors that increased the risk of PPH were maternal age > 35 years (OR: 2.172; 95% CI: 1.206–3.912; *p* = 0.010), preterm delivery < 37 weeks (OR: 5.090; 95% CI: 2.869–9.030; *p* < 0.001) and parity (OR: 1.701; 95% CI: 1.164–2.487; *p* = 0.006). Regarding preterm deliveries, a cesarean section was found to be a risk factor of PPH (OR: 9.310; 95% CI: 1.968–44.057; *p* = 0.005). The comparison of the different characteristics of the case-control analysis is presented in Table 1. Of note, no admissions to the intensive care unit or maternal deaths were recorded following PPH.

Regarding the causes of PPH, uterine atony (“Tone”) was identified in 120 (54.8%) women; placental retention (“Tissue”) was diagnosed in 67 (30.5%) cases, of which 50 (22.8%) involved placenta previa; trauma was identified in 15 women (6.8%); and, finally, a coagulation disorder (“Thrombin”) was diagnosed in three of 17 women with multiple causes of PPH (0.01%). The causes of PPH are presented in Table 2.

Regarding management, 127 patients (57.9%) were treated with uterotonic medication; 99 (45.2%) women did not require any further treatment. In 60 women (27.4%), curettage for an evacuation of the endometrial cavity was needed. In addition, 24 patients (10.9%) undergoing cesarean sections were intraoperatively managed with hemostatic sutures and 16 (7.3%), following a vaginal birth, with suturing of perineal lacerations. In 16 women (7.3%), an obstetric hysterectomy was necessary for the effective management of PPH; overall, during the study period, the rate of hysterectomies was 0.18% (16/8545 deliveries). More specifically, 11 hysterectomies were performed because of a combination of placenta previa and abnormal placentation (accrete, increta or percreta). Three cases were because of uterine atony and two cases because of abnormal placentation alone. Notably, multiple treatment modalities were used in 24 (10.9%) of the above-mentioned cases. A blood transfusion was necessary for 15 women (6.8%).

Furthermore, we performed a subgroup analysis of cases of PPH that were only pharmaceutically (conservatively) treated (n = 99; 45.2%). It was found that preterm delivery and the mode of delivery had a statistically significant impact on the management. Preterm delivery (OR: 2.162; 95% CI: 1.138–4.106; *p* = 0.019) and delivery via a cesarean section (OR: 4.279; 95% CI: 1.921–9.531; *p* < 0.001) were associated with a higher need for additional measures of treatment (Table 3).

Regarding obstetric hysterectomies, an advanced maternal age (OR: 4.485; 95% CI: 1.552–12.960; *p* = 0.006), preterm delivery (OR: 11.099; 95% CI: 3.049–40.409; *p* < 0.001) and parity (OR: 9.556; 95% CI: 1.238–73.776; *p* = 0.030) increased the risk of this debilitating operation in the univariate analysis. Notably, the multivariate analysis confirmed only prematurity as an independent risk factor (OR: 8.695–2.324–32.527; *p* = 0.001) (Table 4).

As for the need for a blood transfusion following PPH, a maternal age > 35 years (OR: 3.005; 95% CI: 1.035–8.729; *p* = 0.043), prematurity (OR: 4.279; 95% CI: 1.470–12.459; *p* = 0.008) and cesarean delivery (OR: 11.635; 95% CI: 3.517–11.635; *p* < 0.001) were associated with an increased risk in the univariate analysis. However, in the multivariate analysis, only delivery via a cesarean section (OR: 8.884; 95% CI: 2.451–32.204; *p* < 0.001) was identified as an independent risk factor for a blood transfusion following PPH (Table 5).

## 4. Discussion

### 4.1. Main Findings

The principal findings of this study were as follows: (1) during a 7-year period, the incidence of PPH in our tertiary hospital was 2.5%; (2) uterine atony was the primary cause of PPH and placental retention with or without placenta previa was the second most common cause of PPH; (3) an advanced maternal age, preterm delivery and parity were the major risk factors for PPH; (4) prematurity and cesarean sections were associated with an increased risk of non-conservative (non-medical) management; (5) preterm delivery was an independent predictor for obstetric hysterectomies; and (6) delivery via a cesarean section was identified as an independent risk factor for blood transfusions following PPH.

### 4.2. Interpretations of the Findings

In a pregnant population of Greece, PPH affected about 1 in 40 pregnancies, which was in accordance with previously published data [1]; other studies have reported an incidence of severe PPH (>1000 mL) of about 0.5–2% [13]. Of note, the PPH rate may increase up to 20% if blood loss is objectively quantified [14]. Almost 150,000 women die every year because of PPH worldwide in low–middle- and high-income countries [14]. Notably, no maternal deaths or admissions to an intensive care unit were reported in the study period.

Furthermore, we found that the two main causes of PPH in our population were uterine atony and placental retention. Uterine atony has previously been reported as the main cause of PPH; however, trauma has generally been reported as the second most common cause [3]. This discrepancy may be related to the high rate of cases of placenta previa (about 1 in 5 cases of PPH); these are managed at our tertiary center following referrals from rural hospitals. Notably, the incidence of placenta previa was about 0.5% in the total sample, which was in accordance with published data, with a reported prevalence of placenta previa of about 5.2 cases/1000 pregnancies, whereas the prevalence of major placenta previa was 4.3/1000 pregnancies [15]. Moreover, there are great regional and geographical variations in the prevalence of placenta previa, with the highest rates reported in Asian studies (12.2/1000) and the lowest in European studies (3.6/1000) [15].

With regard to the risk factors for PPH, a maternal age > 35, a prior history of cesarean delivery, a history of uterine surgery, uterine fibroids, chronic hypertension, placenta previa and preterm delivery have been strongly associated with severe PPH [16]. Additionally, the incidence of PPH increased from 0.3% in nulliparous women to 2% in high-parity cases (>4) [17]. Interestingly, the induction of labor and delivery via a cesarean section, irrespective of a history of previous cesarean sections, were not statistically significant factors of PPH in this study; the induction of labor is reported to be a risk factor for PPH in the literature [18]. The evidence-based use of prostaglandins and oxytocin as per protocols in labor inductions may potentially be the reason why the induction of labor was not a significant factor for PPH in the present study. Of note, a cesarean section was found to be a significant risk factor for PPH in preterm deliveries in our sample; this could be attributed to the fact that placenta previa with or without abnormal placentation was the main reason to perform a preterm cesarean section.

According to our findings, preterm delivery and cesarean sections were associated with an increased risk of non-conservative management. This could be attributed to the fact that most cases of premature deliveries were iatrogenic due to placenta previa or women with multiple previous cesarian sections where the effect of multiparity on non-conservative management was eliminated by the mode of delivery. Of note, a high parity has previously been associated with an increased risk of uterine atony not responsive to medical treatment [17], while other risk factors for PPH not responsive to medical treatment have not been identified in the recent literature.

Furthermore, 0.18% of the participants underwent an obstetric hysterectomy during the study period, while prematurity was strongly associated with obstetric hysterectomies. According to the literature, the risk factors for an emergency postpartum hysterectomy are a maternal age > 35 years, a cesarean section (either a previous or the present delivery), vaginal birth after a previous cesarean delivery (VBAC), preterm delivery, high parity, placenta previa and abnormal placentation [19]. Of note, no cases of obstetric hysterectomies after VBAC were reported, possibly due to the low rate of trials of labor after cesarean deliveries performed in the department (about 1%). A cesarean hysterectomy at the late preterm period was the most common management option in women presenting with placenta previa plus abnormal placentation (placenta accreta, increta and percreta), which was in accordance with the literature [16,17]. According to our local guidelines, the primary management option for placenta previa is the intraoperative management of PPH with different suturing techniques, while a cesarean hysterectomy is usually the last choice in cases of unsuccessful bleeding control. Of note, in all cases undergoing a hysterectomy, abnormal placentation or placenta previa were confirmed following a histopathological examination.

A cesarean section was identified as an independent risk factor for blood transfusions following PPH. This was in accordance with previously published data; factors associated with blood transfusions following PPH were a maternal age > 35 years, prematurity, a cesarean section, placenta previa, twin pregnancy, HELLP syndrome and small-for-gestational-age neonates [18].

With regard to the prevention of PPH, the current guidelines recommend the administration of prophylactic uterotonics after the delivery of the neonate [2]. The active management of the third stage of labor is also strongly recommended as a means to reduce the incidence of PPH; according to the Cochrane review by Begely et al., the active management of the third stage compared with expectant management was associated with decreased blood loss, the need for a transfusion and the therapeutic use of uterotonics [20]. Of note, the active management of the third stage of labor was part of our department’s protocol during the whole study period.

To our knowledge, this is the first study reporting on the epidemiology of PPH in the Greek population. The identification of the risk factors for PPH, the recording of these cases and their management could contribute to a better control and recognition of the situation. The main limitations of the study were its retrospective nature and the fact that many high-risk pregnancies in Northern Greece are referred to our tertiary hospital for antenatal surveillance and delivery. Additionally, the small sample size could justify the wide CIs reported in the analyses.

## 5. Conclusions

The incidence of PPH in a tertiary center was about 1 in 40 deliveries; placental etiology was the second most common cause following uterine atony. Almost half of the cases complicated by PPH were effectively treated with uterotonic medication. An advanced maternal age, prematurity and parity were significant risk factors for PPH. Preterm deliveries and cesarean sections were more likely to require multiple treatments for the management of PPH. Furthermore, prematurity was an independent risk factor for an obstetric hysterectomy. Severe morbidity is uncommon in a tertiary setting, and no maternal deaths due to PPH were reported in the 7-year study period. Currently, the prediction tools for PPH are not implemented in clinical practice as they have not been internally and externally validated. More research on this severe obstetric complication, including the identification of risk factors through the development and validation of prognostic models or prediction tools easily applicable to the general obstetric population, may improve perinatal outcomes.

## Figures and Tables

**Table 1:** Comparison of the different characteristics between the study and control groups.

Parameters	Study Group n = 219 n (%)	Control Group n = 222 n (%)	ORs (95% CI)
**Maternal age**			
>35	37 (16.9)	19 (8.6)	2.172 (1.206–3.912) ^a^
≤35	182 (83.1)	203 (91.4)	Reference
**Gestational age**			
<37	65 (29.7)	17 (7.7)	5.090 (2.869–9.030) ^b^
≥37	154 (70.3)	205 (92.3)	Reference
**Mode of delivery in prematurity** **(<37 weeks)**			
Cesarean section	36 (55.4)	2 (11.8)	9.310 (1.968–44.057) ^c^
Vaginal delivery	29 (44.6)	15 (88.2)	Reference
**Parity in prematurity ** **(<37 weeks)**			
Parous	18 (27.7)	8 (47.1)	--
Nulliparous	47 (72.3)	9 (52.9)	Reference
**Parity**			
Parous	137 (62.6)	110 (49.5)	1.701 (1.164–2.487) ^d^
Nulliparous	82 (37.4)	112 (50.5)	Reference
**Onset of labor**			
Spontaneous labor	115 (52.5)	120 (54.4)	--
Labor induction	57 (26.0)	62 (27.9)	--
No labor	47 (21.5)	40 (18.1)	Reference
**Mode of delivery**			
Cesarean section	50 (22.8)	42 (18.9)	--
Vaginal delivery	169 (77.2)	180 (81.1)	Reference

OR: odds ratio; CI: confidence interval. Only statistically significant associations are presented. ^a^ *p* = 0.010; ^b^ *p* < 0.001; ^c^ *p* = 0.005; ^d^ *p* = 0.006.

**Table 2:** Causes of postpartum hemorrhage.

Causes of PPH	n (%)
Tone Uterine atony	120 (54.8)
Tissue Placental retention Combination Placenta previa ± Abnormal placentation (accreta, increta and percreta)	67 (30.6) 17 (7.8) 50 (22.8)
Trauma Perineal ± vaginal ± cervical	15 (6.8)
Multifactorial Atony ± trauma ± blood disorders	17 (7.8)
Total	219 (100)

PPH: postpartum hemorrhage.

**Table 3:** Subgroup analysis of conservative management only vs. other management options of postpartum hemorrhage.

Parameters	Conservative Management (n = 99) n (%)	Other Management (n = 120) n (%)	Univariate Analysis ORs (95% CI)	Multivariate Analysis ORs (95% CI)
**Maternal age**				
>35	26 (26.3)	27 (22.5)	--	--
≤35	73 (73.7)	93 (77.5)	Reference	Reference
**Gestational age**				
<37	20 (20.2)	50 (41.7)	4.993 (2.717–9.178) ^a^	2.162 (1.138–4.106) ^b^
≥37	79 (79.8)	70 (58.3)	Reference	
**Parity**				
Parous	64 (64.6)	73 (60.8)	--	--
Nulliparous	35 (35.4)	47 (39.2)	Reference	Reference
**Onset of labor**				
Spontaneous labor	63 (63.6)	52 (43.4)	--	--
Labor induction	30 (30.3)	27 (22.5)	--	--
No labor	6 (6.1)	41 (34.2)	Reference	Reference
**Mode of delivery**				
Cesarean section	9 (9.1)	41 (34.2)	5.190 (2.374–11.346) ^a^	4.279 (1.921–9.531) ^a^
Vaginal delivery	90 (90.9)	79 (65.8)	Reference	Reference

OR: odds ratio; CI: confidence interval. Only statistically significant associations are presented. ^a^ *p* < 0.001; ^b^ *p* = 0.019.

**Table 4:** Subgroup analysis of obstetric hysterectomy vs. other management options of postpartum hemorrhage.

Parameters	Obstetric Hysterectomy (n = 16) n (%)	Other Management (n = 203) n (%)	Univariate Analysis Ors (95% CI)	Multivariate Analysis Ors (95% CI)
**Maternal age**				
>35	9 (56.3)	45 (22.2)	4.485 (1.552–12.960) ^a^	--
≤35	7 (43.7)	158 (77.8)	Reference	Reference
**Gestational age**				
<37	13 (81.2)	57 (28.1)	11.099 (3.049–40.409) ^b^	8.695 (2.324–32.527) ^d^
≥37	3 (18.8)	146 (71.9)	Reference	Reference
**Parity**				
Parous	15 (93.8)	124 (61.1)	9.556 (1.238–73.776) ^c^	--
Nulliparous	1 (6.2)	79 (38.9)	Reference	Reference
**Onset of labor**				
Spontaneous labor Labor induction	0 0	115 (56.6) 57 (28.1)	-- --	-- --
No labor	16 (100)	31 (15.3)	Reference	Reference
**Mode of delivery**				
Cesarean section Vaginal delivery	16 (100) 0	34 (16.7) 169 (83.3)	-- Reference	-- Reference

OR: odds ratio; CI: confidence interval. Only statistically significant associations are presented. ^a^ *p* = 0.006; ^b^ *p* < 0.001; ^c^ *p* = 0.030; ^d^ *p* = 0.001.

**Table 5:** Subgroup analysis concerning the need for blood transfusion after postpartum hemorrhage.

Parameters	Blood Transfusion n = 15 n (%)	No Blood Transfusion n = 204 n (%)	Univariate Analysis Ors (95% CI)	Multivariate Analysis Ors (95% CI)
**Maternal age**				
>35	7 (46.7)	46 (22.5)	3.005 (1.035–8.729) ^a^	--
≤35	8 (53.3)	158 (77.5)	Reference	Reference
**Gestational age**				
<37	8 (53.3)	43 (21)	4.279 (1.470–12.459) ^b^	--
≥37	7 (46.7)	161 (79)	Reference	Reference
**Parity**				
Parous	11 (73.3)	127 (62.2)	--	--
Nulliparous	4 (26.7)	77 (37.8)	Reference	Reference
**Onset of labor**				
Labor induction	1 (6.7)	56 (27.4)	--	--
Spontaneous labor	3 (20)	112 (55)	--	--
No labor	11 (73.3)	36 (17.6)	Reference	Reference
**Mode of delivery**				
Cesarean section	11 (73.3)	39 (19.1)	11.635 (3.517–38.489) ^c^	8.884 (2.451–32.204) ^c^
Vaginal delivery	4 (26.7)	165 (80.9)	Reference	Reference

OR: odds ratio; CI: confidence interval. Only statistically significant associations are presented. ^a^ *p* = 0.043; ^b^ *p* = 0.008; ^c^ *p* < 0.001.

## Data Availability

Data are available upon request.
